# The complexity of human papilloma virus in cancers: a narrative review

**DOI:** 10.1186/s13027-023-00488-w

**Published:** 2023-02-26

**Authors:** Vahideh Hamidi Sofiani, Parsa Veisi, Mohammad Rezaei Zadeh Rukerd, Reza Ghazi, Mohsen Nakhaie

**Affiliations:** 1 Department of Microbiology, Golestan University of Medical Sciences, Gorgan, Iran; 2 Gastroenterology and Hepatology Research Center, Institute of Basic and Clinical Physiology Sciences, Kerman University of Medical Sciences, Kerman, Iran; 3 Department of Biotechnology, Golestan University of Medical Sciences, Gorgan, Iran

**Keywords:** Human papilloma virus, Oncoprotein, Cancers

## Abstract

Among human tumorigenic viruses, the role of Human papillomavirus (HPV) has been proven as one of the most important oncoviruses that are associated with a large number of cancers. Most cancers of the genital area such cervical and anal cancer as are caused by HPV, and in many other cancers, such as colorectal, gastric, liver, esophageal, urinary bladder, and head and neck cancers, it is considered as one of the important risk factors. Our search was conducted for published researches between 2000 and 2022 by using several international databases including Scopus, PubMed, and Web of Science as well as Google scholar. We also evaluated additional evidence from relevant published articles. It has been demonstrated that HPV can promote tumorigenesis via focusing on genes, proteins, and signaling pathways, by using E6 and E7 oncoproteins and inhibiting two crucial tumor suppressors, P53 and Rb. The following study was performed to investigate different malignant cancers under the influence of HPV infection and changes in molecular factors caused by HPV infection.

## Introduction

Cancer ranks as the first or second leading cause of death before the age of 70 in more than half of the world's countries, according to a World Health Organization (WHO) report in 2019 [1]. Therefore, the global burden of cancer incidence and mortality is rising quickly [1].

It is estimated that infectious agents cause up to 50% of all human cancers, with viruses accounting for 10–15% of all cases [2]. Many environmental factors, such as viruses, can cause cell destruction, premalignant lesions and neoplasm. The main known viruses that cause cancer in humans include hepatitis B and C viruses, human herpes virus-8 (HHV-8), human T lymphotrophic virus type 1 (HTLV-1), Epstein–Barr virus (EBV), and human papillomavirus (HPV) [3].

HPV is linked to nearly 5% of all cancers worldwide [4]. HPV is the most common sexually transmitted infection, as well as one of the few viruses that can cause multiple benign or malignant cancers in more than half million individuals annually [5, 6]. HPV is associated with a variety of serious cancers, such as gastrointestinal, cervical, urinary bladder, and head and neck cancers. These cancers are becoming a global concern and are responsible for the majority of cancer deaths in developing countries [7]. Currently, researchers are investigating the effects of viral agents on cancer. HPVs are potentially associated with carcinogenesis in various cancer categories. The current study was designed to investigate HPV oncogenesis and the role of HPV in various cancers.

## HPV oncogenesis

HPV is small and non-enveloped virus with double-stranded circular DNA [6, 8]. HPV genome is divided into three regions: 1. The non-coding region, which affects the replication and transcription 2. The early region, which encodes E1, E2, E4, E5, E6, and E7 proteins 3. The late region, which encodes L1 and L2 as capsid proteins. E6 and E7 proteins of the early region have an essential role in the oncogenic properties of HPV [9].

The HPV life cycle is related to keratinocytes, which are found in the epidermis and squamous epithelium of the genitals, oral cavity, and esophagus. New virions of HPV are observed in keratinocytes under differentiation [10]. Epithelial trauma promotes HPV entry into basal epithelial cells and maintains the viral episome in the infected cells [11]. The first reports of HPV-induced cell transformation appeared in the 1980s; HPV-induced cell transformation was caused primarily by E6 and E7 proteins[12].

**Fig. 1:**
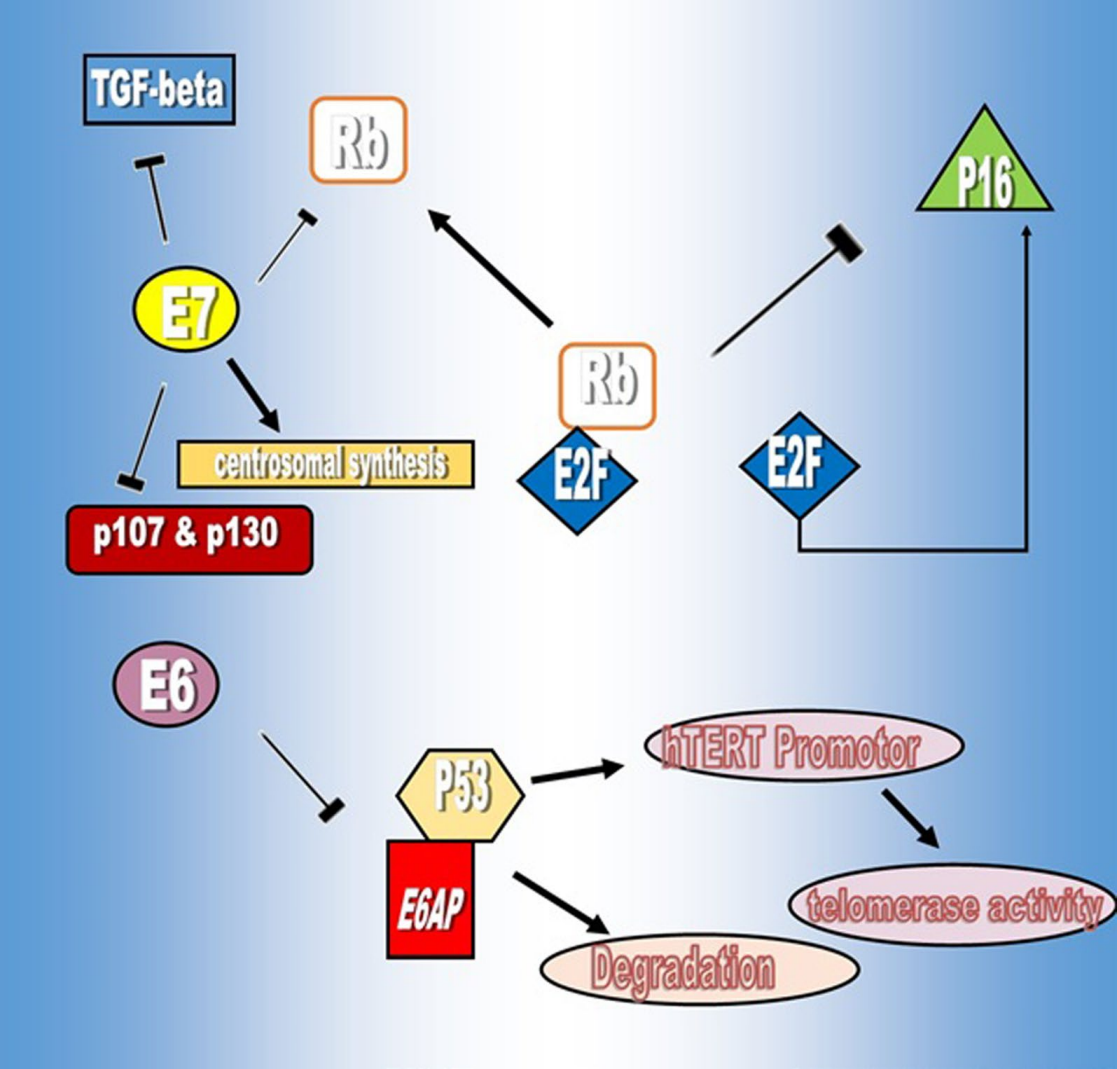
E6 activity VS E7 activity

E6 and E7 promote excessive cell cycle proliferation by interfering with regulatory proteins such as p53 and pRb [13] (Fig. 1). p53 is a tumor suppressor protein that regulates cell proliferation under various conditions. E6 inhibits p53 with different pathways, such as affecting E6AP, a ubiquitin ligase that stimulates proteasome-dependent induced degradation [14], which requires the construction of an E6/E6AP/p53 complex to facilitate the degradation of p53 [15]. Another way E6 inactivates p53 is through its inhibitory effect on the p53 co-activator, known as p300/CBP [16]. p300/CBP are strictly homologous of acetyltransferases, which are enzymes that responsible for histone 3 lysine 27 acetylation (H3K27ac) at the regulatory regions of genes such as enhancers and promoters and are essential factors in the activation of p53 [17].

Furthermore, the presence of the C-terminal PDZ-binding motif (PBM) of the E6 protein indicates the high oncogenic potential of this protein that grants interplay with the PDZ domain of other suppressor proteins such as MAG1,2,3, MUPP1, TIP2/GIPC and affects their standard regulatory properties [18, 19]. E6 oncoprotein also dysregulates multiple regulatory pathways, such as apoptosis and senescence. The interaction of HPV16 E6 with the cellular protein NFX1-123 promotes the expression of human telomerase reverse transcriptase (hTERT), the telomerase catalytic subunit. Due to high telomerase activity, cellular senescence is delayed [20]. In addition, NFX1–123 enhance the levels of the Notch 1 pathway for upregulation of its target genes, such as the differentiation markers to modify keratinocyte differentiation [21].

Another function of E6 is the protection of affected cells from tumor necrosis factors. These factors induce apoptosis by binding to the C-terminal region of tumor necrosis factor, and the E6 protein plays its inhibitory role by preventing the transmission of the apoptotic signal. E6 binds to the Fas-associated death domain (FADD) and prevents E6-expressing cells from responding to apoptotic stimulation. Generally, FADD, as an adaptor protein, interacts with the intracellular death domain (DD) of the Fas receptor (FasR) during Fas ligation with Fas ligand (FasL) during apoptosis [22].

E7 is another oncoprotein that targets retinoblastoma protein (pRb) and pocket proteins p107 and p130. pRb is a regulatory protein in cell cycle proliferation. This protein interacts with E2F, a transcription factor, to inhibit the transcription of genes required for the entrance of cells to the S-phase. E7 oncoprotein promotes cell cycle progression by releasing E2F through pRb ubiquitination. The released E2F causes the transcription of cyclin E, cyclin A and p16 factors as Cyclin-dependent kinase 4 and 6 (CDK4/6) inhibitors and cell entry into the S phase [23]. E7 can also inhibit the effect of p21 and p27, an inhibitor of CDK, to promote cell proliferation [24]. Furthermore, E7 leads to abnormal centrosome synthesis apart from cell division. The expression of HPV-16 E7 induces abnormal centrosome duplication in cells lacking functional pRB, p107, and p130. These results demonstrate that the molecular mechanism whereby HPV-16 E7 induces centrosome duplication errors is independent of its ability to inactivate pRB, p107, and p130 or to interact with the S4 proteasome subunit that causes chromosome missegregation and aneuploidy [25]. Centrosomes are microtubule-organizing centers in human cells that are required for mitotic spindle formation. Centrosome abnormalities in HPV-associated tumors cause mitotic defects and aneuploidy [26].

Another target of E7 in neoplastic cells is transforming growth factor (TGF)-beta through restriction of TGF-beta anti-proliferative effects and develops resistance to TGF-beta. Disruption of the TGF-beta pathway through cancer might contribute to malignant progression [27]. Aside from the previously mentioned properties, both E6 and E7 proteins target the C-myc oncogene, which has been identified as a marker for several types of cancers. C-myc is a transcription factor that regulate cell growth and differentiation. Under the influence of two E6 and E7 oncoproteins, C-myc protein disrupts cell progression, apoptosis, and transformation.

These oncoproteins are under the control of the E2 protein. The E2 proteins are the major transcriptional regulators of the papillomaviruses. The E2 proteins function primarily by recruiting cellular factors to the viral genomes that activate or repress transcriptional processes. E2 protein functions are occasionally interrupted by mutation or integration of the viral genome. This inactivation resulted in reduced E2-mediated repression and enhanced expression of the E6 and E7 genes [28].

Furthermore, HPV oncoproteins target epigenetic changes such as microRNA (miR) expression, which affects the expression of this non-coding RNA (microRNAs) [29] (Table 1).

**Table 1:** Oncogenic properties of HPV oncoproteins

Oncoproteins	Mechanisms	Effects	References
E6	I. Binding to cellular ubiquitin ligase E6-associated protein (E6-AP) II. Binding to co-activator of p53	Promotion of p53 degradation	[14]
	Binding to the cellular proteins containing (PDZ) domains	degradation of potential tumor suppressor proteins	[18]
	Binding to the Fas-associated Death Domain (FADD)	Prevention of Fas-induced Apoptosis	[22]
	Binding to myc proteins and inducing expression of the human telomerase reverse transcriptase (hTERT) catalytic subunit	Telomerase activation leads to prolonged life of HPV-16 infected cells	[20]
	Binding to NFX-1 isoforms: NFX1-123	Increase in hTERT mRNA levels	[30]
E7	I. Proteasomal degradation of the pRB/E2F repressor complex II. Binding to p107 and p130	Activation of genes necessary for S-phase progression	[24]
	Abrogation of the inhibitory activities of the p16 and p27	Dysregulation of the G1/S-phase transition	[31]
	Inhibition of TGF-β signaling	Impaired cellular differentiation	[27]

## Gastrointestinal cancers

### Colorectal cancer

Colorectal cancer (CRC) is the third most common cancer worldwide. CRC is the third and second most common type of cancers in men and women, respectively [32]. Adenocarcinomas are responsible for nearly 95% of all CRC [33]. CRC is caused by a variety of risk factors, including genetic and environmental factors. Viruses, particularly HPV, are an environmental factor in CRC formation. HPV can infect colorectal cancer in a variety of ways, including anogenital, hematogenous and lymphogen transmission, sexual behavior, and vertical transmission from the mother to fetus. HPV vaccination has even been shown to reduce the risk of colorectal cancer in young people. The adjacent normal tissues, including endothelial cells, lymphocytes and fibroblasts, are where oncoprotein E6 has the highest expression. The prevalence of the virus isolated in colorectal cancer is 0 to 84%, with the highest prevalence in South America, Asia and the Middle East. The lowest prevalence is observed in North America. The most isolated types of HPV, which identified were 16, 18, 31, 33, and 45. The prevalence of 16 and 18 was reported to be higher than others among these types [34].

This pathogenesis should be investigated at the molecular level to better understand the etiology of virus-associated cancers. The integration of viral DNA into the cellular genome results in partial or complete loss of the E2 gene, which leads to increased expression of the HPV E6 and E7 oncogenes. Overexpression of E6 and E7 oncoproteins leads to the degradation of p53 and pRb, inducing HPV carcinogenesis [35].

Adenomatous polyposis coli (APC) is a tumor suppressor gene, which widely mutated in CRC. Early occurrence of colorectal tumorigenesis was observed during the mutation of APC gene. The APC pathway is important in the initiation and progression of CRC carcinogenesis. The APC gene inhibits the transition from the G0/G1 to the S phase of the cell cycle. Moreover, cyclin D1 (CCND1) is another target in the APC pathway, and mutant APC cells activate downstream targets such as CCND1 and Myc [36]. The CCND1 gene encodes the regulatory subunit of a holoenzyme that phosphorylates and inactivates the retinoblastoma protein (pRB), in order to promote cell cycle progression [37].

The CCND1 gene encodes the regulatory subunit of a holoenzyme that phosphorylates and inactivates the retinoblastoma protein and promotes progression through the G1-S phase of the cell cycle [38]. Actually, CCND1, along with other cyclin-dependent kinases inhibitors (such as p27 (CDKN1B) and p21 (CDKN1A) are two important regulators at the G1/S checkpoint and are associated in growth-arrested cells and vital for cell growth and apoptosis as well as cell cycle control, mainly during the transition from G1 to S phase [39]. HPV E7 can dysregulate the cell cycle by binding to several cellular proteins. E7 inhibits CDKs inhibitors, such as p27 (CDKN1B) and p21 (CDKN1A) [40]. The c-MYC gene, located in chromosomal area 8q24, is considered an integration site for the HPV genome [41]. Therefore, it is a crucial oncogene element involved in tumor progression and c-MYC deregulation caused by mutations in APC in CRC [42]. The E6 protein of HPV activates telomerase by upregulation of transcription related to hTERT. E6 causes C-Myc to bind to the hTERT promoter. These binding disrupted hTERT promotor, and telomerase inhibitors continue the activity without restriction [43]. Wnt/catenin and Notch signaling pathways are two of the cellular signaling pathways related to CRC. Catenin interacts with various notch pathway members, which are important cell differentiation regulators and play a role in CRC carcinogenesis. Notch, on the other hand, is thought to be a tumor suppressor pathway that is affected by E6 in order to limit its suppressor effect [44]. As a result, despite epigenetics' effect on CRC carcinogenesis, HPV infection duplicated similar effects and affects malignant conditions.

### Anal cancer

Anal cancer, which is classified into two types: squamous cell carcinomas and non-squamous cancers [45], accounts for 2 to 3% of all gastrointestinal tumors [46]. Anal cancer, which is generally considered rare, has been increasing in recent years [46]. Although various factors such as viral infections, cervical tumors, immunosuppression, sexual activity and smoking are involved in its occurrence and development, changes in sexual behaviors, such as increased anal intercourse, especially among men who have same-sex relationships (MSM), may be a contributing factor to the cancer's recent rise [47, 48]. HPV, which is involved in approximately 5% of cancers worldwide [4], can play a role in anal carcinogenesis by penetrating the transformation area in the columnar mucosa of the rectum, distal to the dentate line, and increasing from the squamous junction to the proximal side [49]. So, HPV is associated with approximately 90% of anal cancers with high cytological and histological grade (grades 2 and 3), and interestingly, 70% of these cancers are associated with genotypes 16 and 18, and 80% of them with 16 [46, 50]. However, in the study of Alemany et al., the odds ratio for HPV-16 and HPV-18 positive patients developing anal cancer was reported to be 2 and 7 times, respectively [51]. HPV-39, 56, 59, 66, and 68 subtypes cause low-risk neoplasms in people with condylomata acuminates. However, the presence of low-risk HPV enhances the risk of anal cancer to obtaining high-risk HPV subtypes such as 16, 18, 31, 33, and 45 [52]. The initiation of the oncogenesis process of this virus through E6 and E7 proteins has been proven in many cancers; in the case of anal cancer, this relationship has also been reported by Da Costa et al.'s study that E6 expression as a p53 inhibitor increases the risk of high-grade anal neoplasia [53]. In addition, the increased expression of p16INK4A as a marker of HPV infection in 95% of anal cancers related to HPV DNA is involved in the phosphorylation and inhibition of retinoblastoma protein (Rb) [54].

### Gastric cancer

Gastric cancer is one of the most common, with a high mortality rate that affects men more than women. It should be noted that geographical diversity can be effective in the occurrence of this cancer [55, 56]. Excessive salt consumption, smoking, long-term exposure to nitrosamines in food and water, and microbial infections are all risk factors for gastric cancer [57]. Several studies have highlighted the important role of various infectious agents in gastrointestinal neoplasia, including Helicobacter pylori (H. pylori), EBV, and HPV. Currently, according to various studies, the relationship between HPV infection and gastrointestinal tract neoplasia has been investigated [58]. There is a chance that the virus will infect the stomach and play a role in tumorigenesis by infecting the anus, colon, and oral-esophagus. In a meta-analysis study conducted in 2016 on 1917 cases of gastric cancer from 30 studies, the prevalence of HPV was reported to be 28% among these patients, and types 16 and 18 were reported as the most common types in these patients, with 21% and 7%, respectively. Among these 30 studies, the Polymerase chain reaction (PCR) method was the favorite technique of the researchers to identify HPV, so 26 studies used the PCR method, and only four used the In Situ Hybridization (ISH) technique. In terms of histology, the more significant presence of HPV in poorly differentiated/undifferentiated tumors compared to well-differentiated/moderately differentiated tumors and, on the contrary, the lower presence of the virus in advanced tumor stages (TNM III/TNM IV) compared to (TNM I/TNM II) indicates that HPV plays an unclear role in cancer prognosis. However, there is a possibility that repeated infection with this virus in the early stage and progression of the initial stages of the tumor, such as dysplasia or adenocarcinoma in situ, as precursor lesions, played a role [59].

Considering the inhibitory effect of two oncoproteins, E6 and E7, on P53 and Rb genes, in the investigation of the carcinogenesis of this virus in gastric cancer from a molecular aspect, understanding the role of the association of this virus with P53, P16, and P21 genes is particularly important [60–62].

P53 as a tumor suppressor in many HPV-related cancers is targeted and suppressed by the E7 oncoprotein. However, based on previous research, this relationship has not been found to be significant in gastric cancer. Nevertheless, because of the association between HPV-positive gastric cancer samples and p53 mutations, there is a possibility that the virus can play a role in the early stages of carcinogenesis by increasing p53 mutations, as reported in the Anwar study [60].

P21, as a tumor suppressor gene, is downstream of the P53 gene, so there is a possibility that the inhibitory function of this gene is also affected by mutation in the p53 gene. In Jiliang's study, a synergistic relationship was observed between the presence of HPV16 and p21 gene mutation in gastric cancer, in which case the inhibitory function of this gene is disturbed and leads to cancer progression [61]. Unlike the previous two genes, there is a direct relationship between the HPV and p16 in gastric cancer. This gene, partially considered a marker of HPV infection, by inhibiting the phosphorylation of Rb, prevents its tumor suppressor activity. On the other hand, the activity of E7 oncoprotein in inhibiting Rb increases the expression of p16, which events can help the tumorigenesis of the virus through the E7 protein [62].

### Liver cancer

Primary liver cancer is the sixth most common cancer worldwide and the third main reason for cancer death worldwide in 2020 [1, 63]. Liver cancer is divided into primary and metastatic liver cancer [64]. Primary liver cancer is the second most expected malignancy worldwide [65]. Several studies mentioned the correlation of carcinogenesis between HPV and hepatitis C virus (HCV) [66, 67]. It has been reported that the interaction of the HCV nonstructural protein 5B (NS5B) with the HPV oncoprotein E6 causes proteasomal degradation of pRb in liver cancer [66]. However, another study mentioned that HPV was not related to HCC risk. Thus, Among chronic hepatitis C patients older than 18, those with HPV infection were affiliated with a lower risk of hepatocellular carcinoma (HCC) [66]. In 1992 it was suggested that HPV affects HCC so that infection with HPV-16 or HPV-18 subtypes could act simultaneously with hepatitis B virus (HBV) to cause HCC development [68]. According to the results of this study, HPV may be found as a cofactor simultaneously with HBV infection in the onset of the HCC. Both HBV and HPV are DNA viruses that share a replication strategy, such as reverse transcriptase with the integration of viral DNA into the host genome. These viruses may alter the viral integration event or participation in further carcinogenesis by integrating into the hTERT gene in non-random sites. In addition, the site of virus integration in hTERT determines the tumor phenotype [69].

### Esophageal cancer

Esophageal cancer is the tenth most common cancer worldwide [1]. Esophageal cancer is a malignancy that affects men three to four times more than women. There are two types of esophageal cancer, Esophageal squamous cell carcinomas (ESCC) and esophageal adenocarcinoma (EAC) [70]. Although there is disagreement about the complexity of HPV in the initiation of esophageal cancer [71], some studies consider HPV as one of the most important oncoviruses involved in esophageal cancer, especially EAC [72]. In these studies, even though the virus can enter the body through various ways, such as skin and mucosal itching and direct contact with the infected birth canal, the main way of entering the esophageal tissue is oral sex [73]. It has been reported that people who have oral sex with a HPV-infected person more than six times increases the risk of developing esophageal cancer [74]. Furthermore, Barrett's esophagus, considered one of the important risk factors in esophageal cancer, has demonstrated that the presence of HPV in this pre-cancerous tissue causes an increase and progression towards EAC. It should be noted that Barrett's esophagus develops as a result of continuous stimulation of the esophagus by gastroesophageal reflux disease (GERD), in which the squamous epithelium tissue of the distal esophagus changes shape to a tissue to resemble the stomach wall [75]. There have also been reports of HPV genome integration into the host genome following chronic infection with this virus, and high levels of E6/E7 mRNA in EAC [76], which is due to the role of oncoproteins E6 and E7 in inactivating p53 and RB, reports indicate a high expression level of p16 and a decrease in Rb as a result of E7 activity in HPV infected patients with EAC [76]. These findings support the role of HPV in the pathogenesis of esophageal cancer. Furthermore, these studies show that HPV is a poor prognostic factor in patients with ESCC, and the association between HPV infection and ESCC causes a poor response to oncological treatment [71]. In contrast, considering the importance of the TP53 aberrations in cancer progression, the results of some studies have not observed this finding; for example, there was no TP53 mutation in patients with HPV-positive EAC in the study of Costa et al. Almost half of HPV-negative patients with EAC tended to display TP53 mutations., and have reported that HPV infection and p53 and p16 expression are probably not prognostic factors in ESCC [77]. However, some studies have proven an association between EAC and high-risk HPVs, but others have rejected this relationship [78].

## Cervical cancer

Cervical cancer is the fourth most common female malignancy worldwide and illustrates the main global health concern [79]. Approximately 90% of deaths related to cervical cancer deaths occurred in low-income and middle-income countries (LMIC). High-risk subtypes of the HPV cause cervical cancer. Screening of HPV and vaccination programs are effective methods for preventing this infection [80]. Squamous cell carcinoma and adenocarcinoma account for nearly 70% and 25% of all cervix cancers caused by HPV infection, respectively. HPV includes more than 120 several types that cause human skin and mucosal infection. Only 13 – 15 of these types of high-risk HPV (HPV-HR) are related to cervical cancers and other malignancies [81]. HPV 16, which has been linked to nearly 50% of cervical cancers worldwide, is an important HPV-HR-type. HPV 18 is the second most common; Therefore, HPV 16 and 18 are associated with two-thirds of cervical cancer and subsets of cancers of the vulva, vaginal, penis, anus, oropharynx, and skin. The oncogenic properties of two proteins, E6 and E7, which interfere with cell regulation and differentiation, distinguish HPV-HR from other HPV types [82].

Cervical cancer relieves further copies of the chromosome arm 3q, which contains the hTERC gene in the 3q26 location [83]. This gene is considered to be a template for telomerase RNA, which are responsible for the repeat sequence, which enhances tandem to the ends of chromosomes to maintain the telomere length. As a result, abnormal hTERC amplification leads to increased proliferation, resulting in cervical tumors [84]. The activation of telomerase is a relatively early event in the progression of cervical carcinogenesis, so the expression of the hTERC has the potential to act as a biomarker for the diagnosis and prognosis of cervical neoplasia. Moreover, the HPV-infected positive ratio and hTERC-amplification positive ratio in cervical cancer growth consequently with increasing levels of dysplasia which means that amplification of hTERC and HPV infection are associated with more advanced cervical intraepithelial neoplasia (CIN) 3 [85]. Finding the hTERC amplification is beneficial in the diagnosis of cervical cancer. Actually, Thinprep cytological test (TCT) and HPV detection are ecclesiastical screening techniques for cervical cancer nowadays. The hTERC amplification may serve as a supplementary test to enhance the specificity [41]. The c-MYC gene, which is found on chromosome 8q24, has been identified as a more frequent integration site for the HPV genome [86]. This gene's expression rises in tandem with HPV amplification, which is related to obtaining malignant phenotype in cervical cells. As a result, c-MYC is considered an important oncogene effective in tumor progression. Therefore, it could be a potential biomarker for cervical cancer [87]. Moreover, another tumor suppressor related to cervical cancer is miR-22. MicroRNAs are non-coding RNAs that are important in down-regulating gene expression in tumorigenic cellular processes [88]. In cervical tissues, miR-22 conversely is correlated with histone deacetylase 6 (HDAC6). HDAC6 was downregulated by miR-22 at the post-transcriptional level by targeting the specific site in 3’UTR. E6 oncoprotein of HPV is likely to be the interface between the two HDAC6 and miR-22, which leads to the downregulation of miR-22 in cervical cancer [89]. Another miR that is affected by E6 is miR-20b. miR-20b causes morphological cell alterations in cervical carcinoma, and HPV E6 with increasing miR-20b levels promotes carcinogenesis [90].

The transcriptional activation function of E2 is needed for HeLa cell growth inhibition as well as for transcriptional repression of the viral E6/E7 promoter. It was previously proposed that transcriptional repression of the E6/E7 promoter results from E2 binding its cognate sites proximal to the E6/E7 promoter and displacing other cellular transcriptional factors [91].

## Urinary bladder cancer

Urinary bladder cancer accounts for 3% of all global cancer diagnoses and is especially common in the developed world. 90% of bladder cancer diagnoses are made in patients aged 55 and up. Among different risk factors such as genetic background and environmental exposure, infectious agents, such as the HPV are considered an undeniable factor in Urinary bladder cancer development [92]. There are two hypotheses related to the association of HPV with urinary bladder cancers. The first stated that there was an anatomical reason. The urethra is considered a reservoir and direct connection of the urinary bladder with the genital area, demonstrating a natural path for viral migration. The second hypothesis is related to the natural epithelial tendency of HPV. Generally, these viruses can infect epithelial cells with a high tissue tendency and tropism for squamous epithelium [11]. Previous research has found that the prevalence of HPV varies greatly in cases of urinary bladder carcinoma [93]. A significant relationship between HPV infection and an aggravated disease outcome and a higher risk of recurrence in patients with bladder cancer has been confirmed in some cases [92]. The urinary bladder epithelium are a target of HPV infection, especially condyloma acuminatum, which is property of HPV infection and has been reported in the bladder [94]. Most studies, regardless of histological subtype, fail to show a clear-cut relationship between HPV and urinary bladder cancer. Furthermore, cell cycle inhibitors are being studied to determine the relationship between p16 and HPV in urinary bladder cancer. It concludes, however, that negative p16 staining may not rule out certain HPV conditions [95].

## Head and neck cancer

Head and Neck Squamous Cell Carcinoma (HNSCC) refers to cancers of the nasopharynx, paranasal sinuses, oral cavity, oropharynx, hypopharynx, and larynx that affect mucosal linings of the upper aerodigestive tract. HNSCC accounts for 650,000 new cases and more than 350,000 deaths each year. HPV has been identified as a new risk factor for these cancers in recent decades. HNSCC outbreaks vary depending on anatomical region and geographical location [79]. Developing HNSCC with an outbreak ratio from 2:1 to 4:1 in Men is more than in women. In addition, this cancer is diagnosed at 50 to 70 years [96]. Furthermore, the number of HPV-positive patients with oropharyngeal tumors is lower than in other head and neck regions, such as the larynx and oral cavity [97]. The epithelium of the tonsillar crypts of the head and neck region contains programmed cell death-1 ligand-1 (PD-L1), and PDL1 is responsible for immune evasion by binding PD-1 receptors expressed by immune system cells. PD-L1 overexpression in tonsils increases the likelihood of persistent HPV infection and tumorigenesis [98]. In HPV-associated head and neck cancers, wild-type p53 is present and mutations occur at a rate of only 10% or less. However, HPV E6 inhibits p53 function by inactivating that as well. Furthermore, there is no deletion of P16 in these tumors. Since HPV E7 inhibited phosphorylated Rb, which controls cell cycling of host cells, control of E2F is inhibited and P16 is overexpressed. P16 is a tumor suppressor gene that encodes a CDK repressor, which inhibits the complex formation of cyclin D1 and CDK 4/6. Cyclin D1 and CDK 4/6 complex promotes cell cycling by releasing E2F via phosphorylation of the Rb protein. In contrast, the Rb protein/E2F complex also suppresses the transcription of P16, so when HPV-E7 inactivates the Rb protein, P16 is overexpressed [99].

Furthermore, in HNSCC, a wide range of TP53 residues could be mutated. These mutations can affect regarding mRNA and protein expression, secondary structure, apoptosis activity, and DNA-binding affinity in different ways. In general, the TP53 mutational profile was thought to be an independent prognostic factor in HNSCC. When comparing wild-type HNSCC patients to those with mutated TP53, survival analysis revealed that patients with one mutation in the TP53 gene had poorer overall survival [100]. The prevalence of p53 mutations differed significantly between virus-unrelated HNSCC and virus-related HNSCC consisting of nasopharyngeal and HPV-positive oropharyngeal carcinomas (48.3% vs. 7.1%) [101].

### Oral squamous cell carcinoma

Oral squamous cell carcinoma (OSCC) is the most common type of head and neck cancers, accounting for more than 90% of all cancers. OSCC has a high mortality rate and prevalence in certain parts of the world, including the Western world, where alcohol consumption is high, so its prevalence varies geographically [102]. Smoking is one of the most common risk factors for this cancer, along with alcohol [102]. Although the relationship of HPV as a potential etiology of OSCC has not been reported very strongly, there have been several reports of the presence of this virus in this cancer, so that in a meta-analysis study published in 2020, its prevalence ranged from 13.4% to 58% in this cancer has been reported, and the presence of high-risk types (16 and 18) was shown to be 2.8 times higher than low-risk types [103]. HPV infections that are transmitted to the oral cavity through oral sex and open-mouthed kissing are probably the first way that the HPV is transmitted to the mouth and causes OSCCs [104]. Most studies in the molecular field have focused on the p53 and p16 proteins, which are affected by virus oncoproteins. Although P53 protein is targeted by E6 and degraded in many cancers, this protein was not reduced in OSCCs in a number of studies. The justification for this event states that because of the increase in mutant p53 in HPV-negative OSCC patients, as well as the fact that this event occurs prior to HPV infection, E6 loses its ability to bind to this mutated protein and cannot degrade it [105].

Although the results regarding the expression of P16 in HPV-positive OSCC patients are contradictory, an increase in the expression of this protein has been reported in several studies as a result of Rb suppression by E7. As a result, this increased expression suggests a strong link between HPV infection and the presence of P16, which has been proposed as a surrogate marker for HPV-HR in a number of cancer studies [105–107]. c-MYC and MLH1 were other genes targeted by the HPV in this cancer, which have been reported to increase and decrease their expression, respectively. The increase in c-MYC expression by E7 results from the inhibition of Rb and E6 to activate telomerase, and this increase in c-MYC expression will eventually be effective in tumor progression [105]. The E2Fs factor, which is activated by the E7 oncoprotein, targets and suppresses MLH1 as a DNA repair enzyme and tumor suppressor [105].

### Oropharyngeal cancer

Oropharyngeal cancer is considered the sixth leading cause of cancer mortality, with oropharyngeal squamous cell carcinoma (OPSCC) accounting for nearly 50,000 incident cases worldwide. OPSCC is less common than other HNSCC [108]. The outbreak of OPSCC is rising in mutuality to the reducing incidence of carcinomas in other subsites of the head and neck, despite the decreased currency of smoking [109]. Furthermore, sexual behavior, such as oral sex partners, is a significant risk factor for HPV + OPSCC [110]. HPV infection, especially HPV-16, is considered an essential factor in the onset of HPV-positive OPSCC [111]. The increased expression of E6 and E7 is almost communicated with the integration of HPV DNA into the host genome; nevertheless, carcinogenesis happens in the absence of integration of HPV. According to whole-genome sequencing of HPV + OPSCCs, viral integration occurs in 74% of patients [112]. In this cancer, like cervical cancer, disruption of another viral gene, E2, which suppresses the expression of E6 and E7, is observed with a poor prognosis [113]. E2 expression, as well as an intact E2 gene, is more common in HPV16 positive oropharyngeal carcinomas than in non-oropharyngeal carcinomas; the presence of an intact E2 gene is associated with higher HPV viral load, higher viral oncogene expression, and improved clinical outcome in oropharyngeal cancer patients compared to patients with a disrupted E2 gene [114]. In the absence of HPV infection, chromatin-modifying enzymes such as lysine demethylases, KDM6A and KDM6B are active and affect gene expression, including derepression of Homeobox (HOX) genes, which are essential regulators of development and are silenced mainly by polycomb group (PcG) proteins [115]. Furthermore, these enzymes cause Rb function inhibition, which has long been known as a critical oncogenic property of host cell epigenetic reprogramming via Rb-independent induction [116]. Inhibition of Rb activity with E7 is considered an important key to the prevention of an oncogene-induced senescence-like response. CDKN2A is one of the PcG-regulated genes. This gene encodes p16, cell-cycle inhibitory proteins that suppress CDK4 and CDK6 activity, which is required to mitigate Rb-mediated inhibition of cell-cycle progression in uninfected cells [117]. The necessity of p16 activity in HPV-transformed cells specifies the utility of this tumor suppressor protein as an essential biomarker for the diagnosis of HPV + OPSCC, as the expression is much less similar to being lost or downregulated than that of a protein with deleterious or neutral effects on tumor cell fitness. Other Genes that contribute to epidermal differentiation consist of ZNF750, KMT2D, EP300, RIPK4, and NOTCH1, mostly mutated in patients with HPV + OPSCC. Notably, these genes are components of the p53 and Rb pathways targeted with E6 and E7, which are essential in mutations of more than 40% of HPV + OPSCCs [118].

### Hypopharyngeal cancer

Hypopharyngeal cancer (HPC), which primarily affects the hypopharyngeal squamous tissue, is a rare cancer that accounts for less than 5% of all HNSCCs [119]. This cancer has the worst prognosis of any HNSCC, with 75% of new cases diagnosed in stages III or IV. Although heavy alcohol and tobacco use are the most common risk factors for this cancer [119], the role of infectious agents such as HPV in this cancer has received little attention. However, in previous studies, the prevalence of HPV in this cancer was estimated to be between 13 and 24%, and HPV-HR were more related to this cancer [120]. Despite conflicting reports regarding HPV infection and the prognosis of HPC, most studies have shown that HPV-positive HPC patients have a better overall survival than HPV-negative HPC patients, and thus a better prognosis [121]. Various methods have been used to identify the HPV in this cancer. At the genome level, PCR and ISH methods have been used to identify the viral DNA, and at the protein level, the IHC method has been used to identify the p16 protein as a marker for the presence of HPV infection [121]. Although p16 is discussed as a replacement marker for HPV infection in HPC, many studies have suggested that this marker should be used as a surrogate marker for HPV infection in HPC. Also, a number of studies have reported longer survival for p16-positive patients than p16-negative patients [121]. P53 gene, which is mutated in the majority of non-HPV-associated HNSCC cancer cases and is associated with increased tumor aggressiveness, has been reported in HPV-negative HPC patients, which can be a justification in Correlation with good prognosis of HPV-positive HPC patients [122].

### Laryngeal cancer

In terms of incidence and prevalence, laryngeal cancer ranks 18th and 22nd in the world, respectively. One-third of all head and neck cancers are caused by laryngeal cancer [123, 124]. Although different types of this cancer such as adenocarcinomas, sarcomas, lymphoma, neuroendocrine and squamous cell carcinoma (LSCC) have been reported, but LSCCs are the most common type of this cancer [125, 126]. Alcohol and tobacco use, as with other head and neck cancers, have been identified as major risk factors for this cancer. The role of HPV as a risk factor in LSCC has become very interesting in recent studies that have investigated the risk factors of this cancer, so that in different studies, the prevalence of this virus in LSCC is 8–83% and an average of 28% have considered [126]. Various reasons, including the difference in the geographical distribution, the quality of the examined sample, and the sensitivity and specificity of the used methods used for the different prevalence percentages have been mentioned in the studies [126]. The presence of HPV DNA in LSCC alone is not enough to prove the tumorigenicity of the virus, and HPV-HR (16 and 18) mRNAs expression, especially E6 and E7, should be shown, because the expression of these mRNAs can cause laryngeal lesions (vocal polyps, lesions before larynx cancer, laryngeal cancer) increases and the cofactor role of this virus along with other tumorigenic factors can be demonstrated [126]. Although it is very easy to identify the genome of HPV by the PCR method, PCR method should be used to detect the mRNA of the virus [127]. Tumorigenic activities of this virus in laryngeal cancer take place through different pathways, which, in addition to the oncogenic role of e6 and E7 in inhibiting P53 and Rb, can be mention to overexpressed or mutated in the RAS proto-oncogene to disrupt cell-membrane signal transduction by p21, which leads to malignant cell proliferation and larynx carcinoma, and stimulation of the expression of epidermal growth factor receptors, Myc and cyclin D1 genes by E7, which play a role in accelerating the movement of cells from G1 to S phase and the cell cycle, and ultimately the development of larynx cancer [126].

**Fig. 2:**
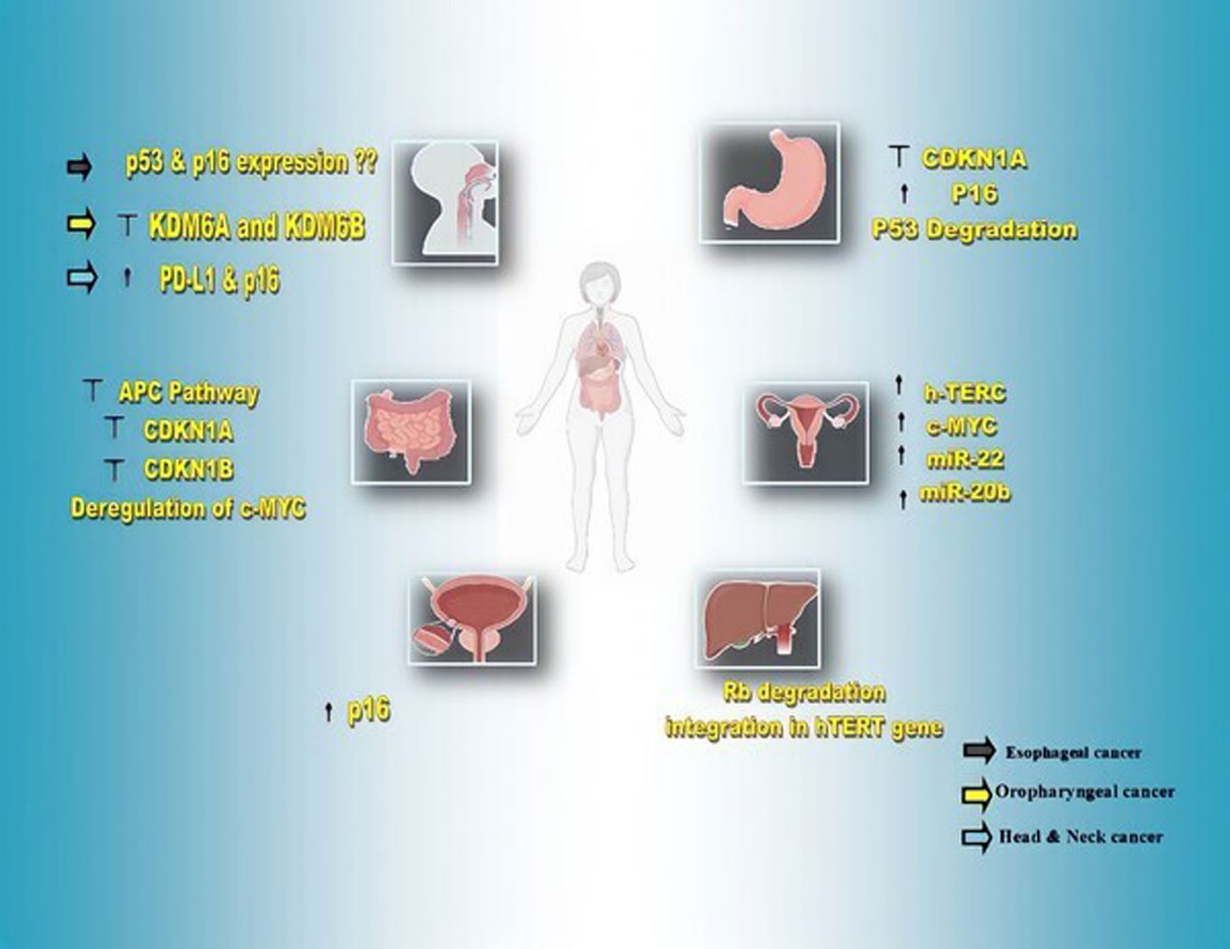
involvement of HPV in molecular markers expression cancers

The expression of P16 protein in this cancer is relatively dependent on the different stages of the cancer, so that its expression is higher in vocal polyps than in benign laryngeal lesions, and in these lesions it is slightly higher than in laryngeal carcinoma [126]. Since the expression of this protein has been reported in 10% of carcinomas, but only in 2% of HPV-positive laryngeal cancer patients, it probably cannot be used as a marker to track HPV infection in this cancer [128]. Of course, according to the data from The Cancer Genome Atlas database (TCGA), which did not report a significant relationship between P16 and the survival of patients with laryngeal cancer, its effect on this cancer is still unknown and needs further studies [126, 129]. In Fig. 2 and Table 2, involvement of HPV in molecular markers expression and detection methods of oncoproteins of HPV-HR-types in various cancers are evaluated, respectively.

**Table 2:** Detection methods of oncoproteins of HPV-HR-types in different cancers

Cancer type	Oncoprotein or Genome	Method	Authors	Year	Description	References
Colorectal & Anal	HPV16 E6	nested-PCR immunohistochemistry	Chen et al.	2012	Virus detection & examination of E6 in colorectal tumors	[130]
Gastric	HPV16 E6	PCR	Ding et al.	2010	Virus detection	[131]
Liver	HPV18 E6, E7	RT-PCR	Tianzhong Ma et al.	2012	Hep G2 cell line contains integrated HPV 18 DNA, leading to the expression of the E6 and E7 oncogenic proteins	[69]
Esophageal	HPV16,18 E6/E7	RNA in-situ hybridization	Rajendra et al.	2017	E6/E7 mRNA transcript analysis	[76]
Cervical	E7	E7 Western blot Immunohistochemistry	Shin MK et al.	2009	p21(Cip1) functions as a tumor suppressor in cervical carcinogenesis and that p21(Cip1) inactivation by HPV-16 E7 partially contributes to the contribution of E7 to cervical carcinogenesis	[40]
Urinary bladder	HPV16,18 E7	Immunohistochemistry	Glenn et al.	2017	Evaluation of HPV E7 oncoproteins expression	[132]
Oral	Different type of HPV	In situ hybridization	Lima et al.	2022	E6 does not bond to P53 due to P53 mutation Increased P16 as a result of E7-mediated Rb suppression	[105]
Oropharyngeal	HPV16 E2	PCR and Real time-PCR	Anayannis et al.	2018	E2 gene is associated with higher HPV viral load, higher viral oncogene expression, and improved clinical outcomes	[11]
Hypopharyngeal	Genome	PCR In situ hybridization	Shi et al.	2022	–	[121]
Laryngeal	HPV16,18 E6/E7	Real time-PCR	Yang et al.	2019	Prove the virus’s presence and tumorigenesis	[126]

## Conclusion and future perspective

During different studies conducted by researchers, the effect of the Human papillomavirus (HPV) on various cancers becomes more pronounced. So, more studies are needed to understand the molecular changes caused by the virus in cancer and to look for an approach to non-invasively sampling from patients with cancers. Many factors are involved in the process of carcinogenesis. And HPVs are considered as one of the important viruses involved in carcinogenesis. Researchers are looking to find markers in the body to detect the changes in the direction of cancer due to these factors. Detecting markers that show the existence of cancers help to improve the treatment and control of cancer before the final stages of the tumor. One of these factors is the p16 protein, one of the inhibitory proteins of cyclin-dependent kinases. Overexpression of this factor in cancers with papilloma viruses is due to the inhibitory effect of E7 on Rb. Another factor is the level of expression of P53, which may be mutated or inhibited. In many cancers, it has been stated that detecting antibodies against P53 can help in the early diagnosis of cancer. Currently, what is considered a cancer-tracking agent is HPV ctDNA. This factor is released into the blood due to the destruction of cancer cells, and researchers believe that identifying this marker can help initiate cancer treatment. According to the studies that have been done, it can be said that knowing the biology of cancer and virus and examining the factors involved in this process can help to obtain ways to control and treat cancers.

## Data Availability

Not applicable.

## Abbreviations

HPV: Human papilloma viruses
WHO: World Health Organization
HHV-8: Human herpesvirus 8
HTLV-1: Human T-lymphotropic virus type 1
EBV: Epstein–Barr virus
H3K27ac: Histone 3 lysine 27 acetylation
PBM: PDZ-binding motif
hTERT: Human telomerase promotor
FADD: Fas-associated protein with death domain
DD: Death domain
FasR: Fas receptor
FasL: Fas ligand
pRb: Retinoblastoma protein
CDK: Cyclin-dependent kinase
TGF-beta: Transforming growth factor-beta
miRNAs: MicroRNAs
CRC: Colorectal cancer
APC: Adenomatous polyposis coli
CCND1: Cyclin D1
MSM: Men who have sex with men
H.pylori: Helicobacter pylori
PCR: Polymerase chain reaction
ISH: In situ hybridization
HCV: Hepatitis C virus
HBV: Hepatitis B virus
NS5B: Nonstructural protein 5B
ESCC: Esophageal squamous cell carcinomas
EAC: Esophageal adenocarcinomas
GERD: Gastroesophageal reflux disease
LMIC: Middle-income countries
HPV-HR: High risk HPV
TCT: Thinprep cytological test (TCT)
HDAC6: Histone deacetylase 6
HNSCC: Head and Neck Squamous Cell Carcinoma
PD-L1: Programmed cell death ligand-1
OSCC: Oral squamous cell carcinoma
OPSCC: Oropharyngeal squamous cell carcinoma
HOX: Homeobox
PcG: Polycomb group
HPC: Hypopharyngeal cancer
LSCC: Laryngeal squamous cell carcinoma
TCGA: The Cancer Genome Atlas database
